# Caspofungin-Induced Cardiotoxicity in Patients Treating for Candidemia

**DOI:** 10.3390/toxics10090521

**Published:** 2022-08-31

**Authors:** Roya Sattarzadeh Badkoubeh, Mostafa Farajpour, Mohammadreza Salehi, Alborz Sherafati, Zahra Zamani, Omid Rezahosseini, Pejman Mansouri, Akram Sardari

**Affiliations:** 1 Cardiology Department, Imam Khomeini Hospital Complex, Tehran University of Medical Sciences, Tehran P.O. Box 14197-33141, Iran; 2 Department of Infectious diseases and Tropical Medicine, Imam Khomeini Hospital Complex, Tehran University of Medical Sciences, Tehran P.O. Box 14197-33141, Iran; 3 Department of Community Medicine, Tehran University of Medical Sciences, Tehran P.O. Box 14155-6559, Iran; 4 Department of Infectious Diseases, Copenhagen University Hospital, Rigshospitalet, 2100 Copenhagen, Denmark; 5 Tehran Heart Center, Tehran University of Medical Sciences, Tehran P.O. Box 14155-6559, Iran

**Keywords:** caspofungin, candidemia, cardiotoxicity, troponin

## Abstract

Echinocandins selectively inhibit fungal cell wall synthesis and, therefore, have few side effects. However, there are reports of hemodynamic and cardiac complications. We conducted this study to investigate the effects of caspofungin both on the noninvasive echocardiographic indices of myocardial function and myocardial injury based on serum high-sensitivity cardiac troponin I (hs-cTnI) levels. This study was conducted on patients treated for candidemia. The hs-cTnI level and echocardiographic parameters were measured before and 1 h after the infusion of the induction dose of caspofungin. Data were compared between central and peripheral venous drug administration routes. Fifteen patients were enrolled in the study. There were no significant differences in the echocardiographic parameters between the baseline and post-treatment period. The mean hs-cTnI level exhibited a significant rise following drug administration (0.24 ± 0.2 ng/mL vs 0.32 ± 0.3 ng/mL; *p* = 0.006). There was also a significant difference concerning the hs-cTnI level between central and peripheral venous drug administration routes (*p* = 0.034). Due to differences in the hs-cTnI level, it appears that the administration of caspofungin may be associated with myocardial injury. Our findings also showed a higher possibility of cardiotoxicity via the central venous administration route.

## 1. Introduction

Echinocandins are deemed efficacious and safe agents in the armamentarium against fungal infections. The mechanism of action of this pharmacological group of fungicides, comprised of caspofungin, micafungin, and anidulafungin, is the inhibition of 1,3-β-D-glucan synthesis in the fungal cell wall [1], which increases the cell wall permeability and leads to cell lysis. Given the absence of cell walls in the human cell structure, echinocandins selectively target fungal cells and are, therefore, associated with not only very few side effects by comparison with azoles and amphotericin B [1] but also with desirable therapeutic properties against candidiasis and aspergillosis. The current guideline of the Infectious Diseases Society of America (IDSA, Arlington, VA, USA) recommends echinocandins as the treatment of choice for candidemia in patients with or without neutropenia and the empirical therapy of suspected candidiasis in patients hospitalized in the intensive care unit (ICU) [2]. Furthermore, echinocandins are used as first-line therapy against Candida endocarditis of the native valve [2].

The major complication of echinocandin use is hypersensitivity, which can also present as anaphylactic shocks [1]. There have also been a few cases of hepatotoxicity. Nonetheless, these complications have been reported in fewer than 10% of all cases of caspofungin use [1]. In recent years, echinocandin-induced hemodynamic complications have also been noted. A case series reported severe hypotension following the administration of echinocandins: one case with caspofungin and two cases with anidulafungin [3]. Two other case reports documented hypotension, bradycardia [4], and flash pulmonary edema [5] after anidulafungin use. The publication of these case series was followed by animal studies aimed at evaluating the effects of echinocandins on cardiomyocytes and cardiac function, which yielded evidence regarding the impact of these fungicides on cardiac hemodynamics [6,7,8]. Nevertheless, to the best of our knowledge, no observational study has yet been conducted to investigate echinocandin-induced cardiotoxicity. We, accordingly, designed the current study to investigate the effects of caspofungin both on the noninvasive echocardiographic indices of cardiac function and myocardial injury based on serum high-sensitivity cardiac troponin I (hs-cTnI) levels.

## 2. Materials and Methods

### 2.1. Study Population

The present interventional study was conducted in Imam Khomeini Hospital Complex between the years 2019 and 2020. The study population was comprised of 15 participants who were being treated with caspofungin at the discretion of infectious disease specialists either for confirmed or suspected candidemia in keeping with the IDSA’s guidelines. The exclusion criteria consisted of concurrent treatment with other cardiotoxic drugs, especially anthracyclines, and a history of heart failure with a left ventricular ejection fraction (LVEF) of less than 50%. Through the use of a questionnaire, the subjects’ demographic characteristics and underlying medical conditions, including a family history of cardiovascular disease and a history of coronary artery disease, hypertension, dyslipidemia, and renal failure, were collected. Additionally, an electrocardiogram (ECG) at baseline was obtained from all the patients to investigate the presence or absence of atrial fibrillation.

In animal studies, the effects of echinocandin use on the heart were dose-dependent and were observed in higher doses [7,8,9]. In the current investigation, the induction dose of caspofungin was selected for the assessment of its effects. The drug was intravenously infused at the dose of 70 mg for 1 h in all the study participants. Cardiac parameters were evaluated once before and once after the infusion.

### 2.2. Echocardiographic and Biochemical Studies

Cardiac function was assessed noninvasively via echocardiography, conducted by a single operator on a GE VIVID E9 machine, prior to caspofungin infusion. The echocardiographic parameters investigated comprised systolic function indices, including LVEF via the eyeball and Simpson methods, the global longitudinal strain, the Tei index, and diastolic function indices, including the ratio between the early mitral inflow velocity and the mitral annular early diastolic velocity (E/E′) and the left atrial volume index. All the patients once again underwent echocardiography 1 h following caspofungin infusion for the measurement of the aforementioned parameters. Additionally, 1 h before and 1 h after the infusion of caspofungin, blood samples were obtained to measure hs-cTnI levels via Elecsys^®^ hs-cTnI immunoassay (Roche, Bazel, Switzerland). The upper level of the normal limit of hs-cTnI was considered to be 0.1 ng/mL. This timeframe was considered based on our interest in assessing the acute hemodynamic effect of the drug, which was reported immediately or within 1 h in previous studies [8,10].

Given the association between echinocandin injection via the central venous route and cardiac effects in animal studies [7,11], in the present investigation, the administration route of caspofungin (peripheral vs central venous administration) was registered for the whole study population to compare the 2 subgroups of administration routes.

### 2.3. Ethical Considerations

The study protocol was approved by the Ethics Committee of Tehran University of Medical Sciences (Code: IR.TUMS.IKHC.REC.1396.3453). Before study enrollment, informed written consent was obtained from all the study patients, who were reassured concerning the confidentiality of their data and were permitted to withdraw collaboration at their own choice.

### 2.4. Statistical Analysis

The statistical analyses were conducted using the SPSS software, version 22.0, and a *p*-value of less than 0.05 was considered statistically significant. The quantitative data were presented as the mean ± the standard deviation, and the qualitative data were presented as numbers and percentages. Due to the small size of the study population, the Kolmogorov–Smirnov test was carried out in the first step to determine whether any distribution was normal or not. Thereafter, based on the results, the 2 variables of eyeball LVEF and E/E′, given their non-normal distributions, were compared using the Wilcoxon rank-sum test, and the other variables were compared using the paired *t*-test.

## 3. Results

The study population was comprised of 15 patients at a mean age of 32 ± 14 years, with the youngest patient aged 22 and the oldest 53. Ten (66.6%) patients were male. Diabetes and hypertension were reported in one patient each. None of the patients had a history of dyslipidemia, renal failure, coronary artery disease, or a family history of cardiovascular disease. ECG showed the sinus rhythm in all the patients; no evidence of atrial fibrillation was detected in any patient’s ECG. Table S1 depicts the characteristics of the patients in the study subgroups.

According to the Kolmogorov–Smirnov test, E/E′ and eyeball LVEF had a *p*-value of less than 0.001 and 0.001, respectively. Consequently, the Wilcoxon signed-rank test was used to compare the values of E/E′ and eyeball LVEF before and after the intervention: no significant difference was observed (*p* = 0.3 and *p* = 0.9, respectively). The *p*-values for the other variables were greater than 0.05. Thus, the paired sample *t*-test was applied to compare the values before and after the intervention for the variables with normal distributions. Our results illustrated that the mean hs-cTnI level before and after the infusion of caspofungin was 0.24 ± 0.2 ng/mL and 0.32 ± 0.3 ng/mL, respectively; the rise constituted statistical significance (*p* = 0.006).

None of the echocardiographic parameters exhibited a statistically significant change following caspofungin infusion compared to its respective baseline value (Table 1). Table S2 reveals the correlations between the variables. The coefficient *r^2^* was used to show the degrees of these correlations. The variables of hs-cTnI and left atrial volume exhibited a significant correlation before and after caspofungin injection (*p* < 0.001 and *p* = 0.02, respectively).

Vis-à-vis the administration route, nine (60%) patients received caspofungin via the peripheral venous route and six (40%) patients through the central venous route. The mean hs-cTnI level showed a 0.17 ± 0.22 ng/mL rise in the peripheral vein subgroup and a 0.53 ± 0.36 ng/mL increase in the central vein subgroup; the difference between the two subgroups was statistically significant (*p* = 0.034).

Table 2 presents the difference between the mean and the standard deviation of the variables between before and after caspofungin injection: only hs-cTnI exhibited a difference of statistical significance with a 95% confidence interval. No statistically significant difference was observed apropos the changes in echocardiographic parameters between the subgroups.

## 4. Discussion

The use of fungicides such as azoles and amphotericin B is associated with cardiac complications. For instance, amphotericin B consumption can cause hypokalemia and increase risk of cardiac arrhythmias, and itraconazole use is accompanied by cardiotoxicity [1]. The literature, however, contains very few human studies on the association between echinocandin use and cardiac complications. Published case reports provide evidence as regards decreased blood pressure and cardiac output following echinocandin infusion [3,4]. One of these reports justified post-echinocandin-use hemodynamic disturbances with the increased release of histamines and anaphylactic reaction to the infusion [5]. Notably, the majority of patients under study in those investigations suffered from cardiovascular disease or renal failure [3,4], rendering them more susceptible to hemodynamic instability. By no means are the cardiac complications of echinocandins limited to those case reports, however. The FDA Adverse Event Reporting System (FAERS) documented 2015 patients with cardiac complications following the consumption of echinocandins between the years 2004 and 2015, with heart failure constituting the most frequent cardiac complication of caspofungin use [12].

Such evidence prompted further animal studies on the effects of caspofungin on cardiomyocytes and cardiac function. Arens et al. [6] observed curtailed contractile responsiveness in isolated cardiomyocytes of rats and reported a dose-dependent increase in the ratio of round-shaped non-contracting cardiomyocytes to rod-shaped normal contracting cardiomyocytes. Koch et al. [7] demonstrated reduced arterial blood pressure and cardiac output in rats receiving high-dose anidulafungin or caspofungin, an effect that was not observed with low-dose anidulafungin or caspofungin. In another study, Koch et al. [8] reported a reduction in arterial blood pressure and cardiac output following the administration of anidulafungin or caspofungin in rats that had received lipopolysaccharides for the simulation of septic shock. In an investigation by Stover et al. [11], caspofungin administration was followed by diminished arterial blood pressure and cardiac output in most of the rats under study, while a few animals exhibited no change.

Few human studies have thus far been undertaken on the hemodynamic effects of echinocandins. Lahmer et al. [10] studied patients under treatment with echinocandins in the ICU and observed a significant drop in diastolic pressure and the mean arterial pressure immediately after drug administration, which was resolved 4 h afterwards; still, none of the patients experienced any change in their cardiac output and systolic blood pressure. This finding may be rationalized by vasodilation, probably due to histamine release. Even so, the results of the current study indicate cardiomyocyte damage following echinocandin administration.

The Fourth Universal Definition of Myocardial Infarction guideline defines myocardial injury as elevated cardiac troponin above the 99th percentile upper reference limit [13]. The measurement of high-sensitive troponin can increase the detection of cardiac injury, as well as lead to earlier detection of damage compared to conventional assays [14]. However, since high-sensitive troponin can be detected in healthy individuals, it should be interpreted quantitatively, i.e., higher levels reflect a greater likelihood of damage [14]. It should also be considered that increased high-sensitive troponin cannot identify the mechanism of injury and can be due to different etiologies [13]. However, elevated high-sensitive troponin in patients receiving cardiotoxic treatment can be helpful in the early detection of myocardial damage in the subclinical phase [15]. Natriuretic peptides are also suggested for screening of cardiotoxicity, although the supporting evidence is not as impactful as troponin [16]. Additionally, the guidelines recommend echocardiography as the primary screening tool, with LVEF <50% or >10% in LEVF as a sign of cardiotoxicity [17]. In summary, the best approach for screening cardiotoxicity is a combination of biomarkers and imaging [16]. Therefore, we decided to assess high-sensitive troponin as well as echocardiography indices.

We observed a significant rise in the mean hs-cTnI level after caspofungin infusion, which may denote cardiomyocyte damage. This finding chimes in with the results obtained in animal studies. Nonetheless, none of the noninvasive indices of systolic and diastolic cardiac indicesexhibited a meaningful change. Considering that hs-cTnI measurement is capable of distinguishing even slight damage to cardiomyocytes and that alterations in echocardiographic indices of systolic and diastolic functions need the involvement of a more extensive area of the heart, our findings may indicate that the extent of the acute injury caused by caspofungin administration is not sufficient to result in acute cardiac dysfunction. Be that as it may, some degree of cardiotoxicity is inevitable, which may be exacerbated by the subsequent doses of the drug and may, in the long term, lead to cardiac dysfunction.

**Figure 1:**
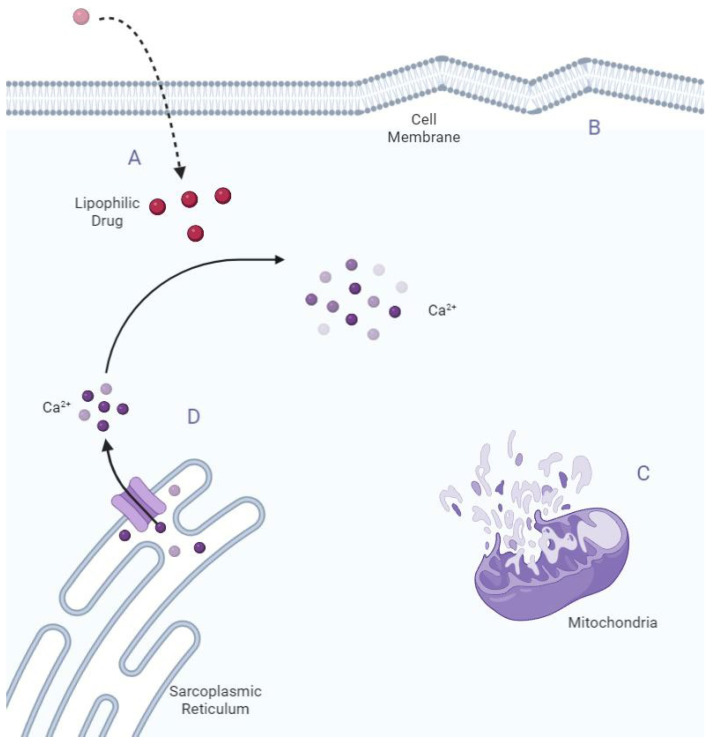
Mechanisms of negative effects of caspofungin on cardiomyocytes. (**A**) Caspofungin lipophilicity can lead to a high concentration in cardiomyocytes; (**B**) Cell member lysis; (**C**) Mitochondrial toxicity; (**D**) Ryanodine receptor activation and increased intracellular calcium release from the sarcoplasmic reticulum.

With respect to the mechanism of echinocandin-induced cardiotoxicity, various hypotheses have been propounded. Taking into account the absence of complications following micafungin, which is a hydrophilic agent, cardiotoxicity may be linked with the lipophilicity of the other two echinocandins [12,18]. It has also been postulated that the disruption of endothelial layers caused by the release of different mediators in sepsis likely augments the cellular permeability of cardiomyocytes and, thus, raises antifungal cell concentrations [6]. Another hypothesis draws parallel lines between the structure of echinocandins and that of surfactin, which can contribute to the lysis of cell membranes [12]. An animal study investigated mitochondrial enzyme activity in cardiomyocytes and refuted the notion of mitochondrial toxicity as the mechanism underlying echinocandin-induced cardiac failure and suggested that calcium homeostasis might drive the pathogenesis [7]. In a study on human cardiomyocytes, caspofungin resulted in increased intracellular calcium concentrations in a dose-dependent manner. This effect was suppressed in the presence of caffeine and ryanodine. Given that both caffeine and ryanodine settle on ryanodine receptors in the sarcoplasmic reticulum, it was postulated that echinocandins could exert part of their cardiotoxicity by the activation of ryanodine receptors (Figure 1) [9]. As was previously mentioned, in animal studies, echinocandin-induced cardiotoxicity is associated with the high dose, as well as the central venous route, administration of this group of antifungal agents [7,8,9,10,11]. Likewise, we found that the central venous administration of caspofungin was associated with a high mean hs-cTnI level by comparison with the peripheral venous route. One probable explanation is delivering a higher concentration of drug to the heart via the central route. It is, therefore, advisable that caspofungin be administered via the peripheral venous route, if possible, to reduce the possibility of side effects.

The salient limitations of the present study are its small sample size which limits statistical power, and the absence of a long-term follow-up, precluding robust conclusions regarding the long-term effects of echinocandin use on cardiac function, including the possible heart failure occurrence. Caspofungin is metabolized by the liver, and its plasma level can be affected by liver enzyme inducer drugs. The level can be increased in critically ill patients in the ICU, especially with low serum albumin [19]. Our patients were young with none of the abovementioned conditions. However, in future studies, chronic liver disease and specific drugs may be considered exclusion criteria. Furthermore, interindividual variability in drug levels based on factors including low serum albumin should be taken into account in any analysis. Because of our inability to transfer old and seriously ill patients from the ICU to the echocardiography ward for a more in-depth echocardiographic study, we enrolled mainly young patients, who generally have few underlying medical conditions that might render them susceptible to cardiac dysfunction. Had we recruited older patients, patients with underlying diseases, or patients hospitalized in the ICU, we might have found more remarkable cardiotoxicity effects, including changes in the echocardiographic indices of cardiac function, following echinocandin infusion. Hence, we suggest that a study on a larger sample volume with a wider age range and more varied underlying medical conditions featuring a follow-up period of longer than 1 year be designed so that it could confer a clearer perspective of echinocandin-induced cardiotoxicity. 

## 5. Conclusions

The results of the present study demonstrated a significant rise in the mean hs-cTnI level following the intravenous infusion of caspofungin; consequently, it appears that the administration of this antifungal agent may be associated with myocardial dysfunction. Our findings also showed a high possibility of echinocandin-induced cardiotoxicity via the central venous administration route in comparison with the infusion through the peripheral venous route.

## Figures and Tables

**Table 1:** Echocardiographic parameters before and after caspofungin infusion.

Parameter	Before	After	*p*-Value
Eyeball LVEF	55 ± 0	54.6 ± 1.2	0.3
Simpson LVEF	55.1 ± 8.6	54.4 ± 6.6	0.79
GLS	−16.2 ± 3.5	−16.5 ± 2.4	0.66
Tei index	0.29 ± 0.07	0.27 ± 0.05	0.47
E/E′	9.13 ± 2.2	9.15 ± 2.6	0.9
LAVI	26.3 ± 9	25.3 ± 6	0.62

LVEF, left ventricular ejection fraction; GLS, Global longitudinal strain; E/E′, ratio between early mitral inflow velocity and mitral annular early diastolic velocity; LAVI, Left atrial volume index.

**Table 2:** Differences in the parameters (mean ± SD, 95% CI) before and after caspofungin infusion.

	N	Mean of the Difference	SD of the Difference	*p*-Value of the Difference	95% CI of the Difference
Simpson LVEF 1&2	15	−0.73	10.68	0.7	−6.6–5.1
GLS 1&2	15	0.38	3.35	0.6	−1.4–2.2
Tei index1&2	15	0.01	0.08	0.4	−0.02–0.06
LAVI 1&2	15	1.01	7.74	0.6	−3.2–5.3
hs-cTnI 1&2	15	−0.11	0.13	0.006	−0.1–0.03

SD, Standard deviation; CI, Confidence interval; LVEF, left ventricular ejection fraction; GLS, Global longitudinal strain; LAVI, Left atrial volume index; hs-cTnI, high-sensitivity cardiac troponin I.

## Data Availability

The data presented in this study are available on request from the corresponding author.

## Supplementary Materials

The following supporting information can be downloaded at: https://www.mdpi.com/article/10.3390/toxics10090521/s1, Table S1: Frequency of the patients in the study subgroups; Table S2: Paired Samples Correlation and the coefficient of determination (before and after caspofungin injection).
