# Supplementary material for: Caspofungin-Induced Cardiotoxicity in Patients Treating for Candidemia
Source: Toxics. 2022 Aug 31;10(9):521. doi: 10.3390/toxics10090521 (PMC9506447; doi:10.3390/toxics10090521)
Supplement: Supplementary file 1 [file toxics-10-00521-s001.zip › toxics-1854860-supplementary.pdf]

Table S1: Frequency of the patients in the study subgroups

		N	%
Gender	Female	5	33
	Male	10	67
Drug infusion	Peripheral	9	60
	Central	6	40
DM	Diabetic	1	6.7
	Nondiabetic	14	93
HTN	Hypertensive	1	6.7
	Non-hypertensive	14	93
Chronic kidney disease	Non-CKD	15	100
Atrial fibrillation	Non-AF	15	100

AF, Atrial fibrillation; CKD, Chronic kidney disease; DM, Diabetes mellitus; HTN, Hypertension

Table S2. Paired Samples Correlation and the coefficient of determination (before and after caspofungin injection)

		N	Correlation	p-value	r^2
Pair 1	Simpson LVEF 1&2	15	0.05	0.8	
Pair 2	GLS 1&2	15	0.41	0.12	
Pair 3	Tei index 1&2	15	0.29	0.27	
Pair 4	LAVI 1&2	15	0.56	0.02	0.31
Pair 5	hs-cTnI 1&2	15	0.92	0	0.85

LVEF, left ventricular ejection fraction; GLS, global longitudinal strain; LAVI, Left atrial volume index; hs-cTnI, high-sensitivity cardiac troponin I
